# Risk Factors and Management of Osteoporosis Post-Transplant

**DOI:** 10.3390/medicina56060302

**Published:** 2020-06-19

**Authors:** Karthik Kovvuru, Swetha Rani Kanduri, Pradeep Vaitla, Rachana Marathi, Shiva Gosi, Desiree F. Garcia Anton, Franco H. Cabeza Rivera, Vishnu Garla

**Affiliations:** 1 Department of Medicine, University of Mississippi Medical Center, Jackson, MS 39156, USA; 2 Division of Nephrology, Department of Medicine, University of Mississippi Medical Center, Jackson, MS 39156, USA; svetarani@gmail.com (S.R.K.); pvaitla@umc.edu (P.V.); dgarciaanton@umc.edu (D.F.G.A.); fcabezarivera@umc.edu (F.H.C.R.); 3 Division of Hospital Medicine, Department of Medicine, University of Mississippi Medical Center, Jackson, MS 39156, USA; rmarathi@umc.edu; 4 Department of Hospital Medicine, Banner Thunderbird Medical Center, Glenadale, AZ 85306, USA; dr.gsky@gmail.com; 5 Department of Internal Medicine and Mississippi Center for Clinical and Translational Research, University of Mississippi Medical Center, Jackson, MS 39156, USA; vgarla@umc.edu

**Keywords:** osteoporosis, organ transplantation, bone disease, kidney transplant, liver transplant

## Abstract

Bone and mineral disorders are common after organ transplantation. Osteoporosis post transplantation is associated with increased morbidity and mortality. Pathogenesis of bone disorders in this particular sub set of the population is complicated by multiple co-existing factors like preexisting bone disease, Vitamin D deficiency and parathyroid dysfunction. Risk factors include post-transplant immobilization, steroid usage, diabetes mellitus, low body mass index, older age, female sex, smoking, alcohol consumption and a sedentary lifestyle. Immunosuppressive medications post-transplant have a negative impact on outcomes, and further aggravate osteoporotic risk. Management is complex and challenging due to the sub-optimal sensitivity and specificity of non-invasive diagnostic tests, and the underutilization of bone biopsy. In this review, we summarize the prevalence, pathophysiology, diagnostic tests and management of osteoporosis in solid organ and hematopoietic stem cell transplant recipients.

## 1. Introduction

Solid organ transplants are increasingly performed for multiple end-stage organ failures [1]. Transplantation has improved longevity and quality of life. Post-transplant osteoporosis can potentially affect morbidity and mortality [2,3]. Consequential reduction in bone density is substantial, especially in the first year post-transplant, and contributes to an increased risk of fractures [4,5]. Significant reductions in bone mineral density are sometimes reported after kidney transplantation, particularly at the lumbar spine [6]. About 22.5% of kidney transplant recipients experience fracture in the first five years after the transplant [7]. With reduced use of glucocorticoids post-transplant, the risk of fracture has decreased tremendously compared to the past [8]. However, fracture risk post-transplant is still higher than it is in the general population [9]. Hence, a thorough knowledge of pre-transplant risk factors, medication side effects and bone morphological changes post-transplant is essential to effectively manage transplant recipients. In this review, we focus on pathophysiology, risk factors, evaluation, diagnosis and the management of post-transplant osteoporosis.

## 2. Bone Morphology

**Figure 1:**
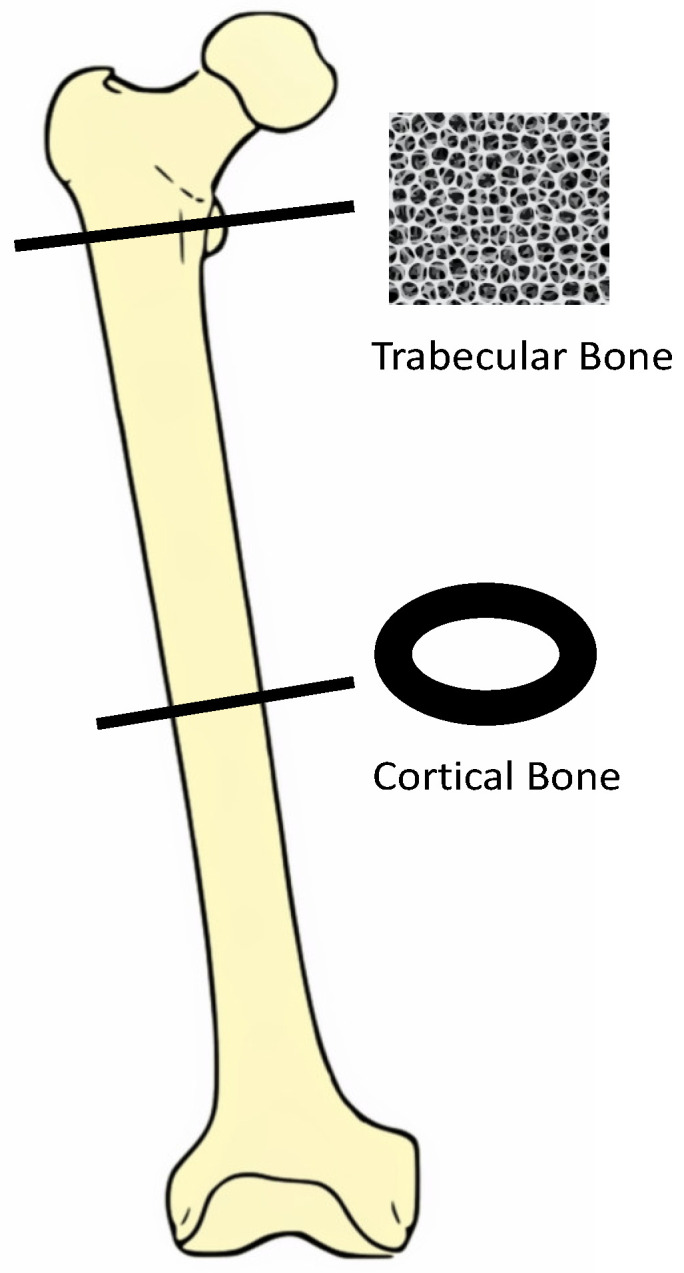
Bone morphology outlined.

Bone is composed of a mineralized collagenous extracellular matrix surrounding a range of specialized cells, including osteoblasts, osteocytes and osteoclasts [10]. The adult human skeleton is composed of 80% cortical bone and 20% trabecular bone [11]. The ratio between cortical and trabecular bone varies with the site [11]. Cortical bone is dense and solid and surrounds the marrow space, while trabecular bone is a honeycomb and extends into the marrow space [10]. Bone remodeling is key to maintaining bone strength; it involves osteoclast-mediated bone resorption, followed by osteoblasts synthesizing the collagenous organic matrix and mineralization of the newly formed matrix [10]. The fragility of bone largely depends on the ratio of osteoblasts (bone-forming cells) to osteoclasts (bone-absorbing cells). In general, the ratio of osteoblasts to osteoclasts decreases with advancing age, leading to bone loss. Bone morphology is detailed in Figure 1.

## 3. Pathogenesis

Osteoporosis is characterized by changes in bone quality and mineralization, which would potentiate fracture risk [12]. In patients undergoing an organ transplant, bone changes evolve through four different phases. First is the presence of bone mineral abnormalities in end-stage organ failure. Secondly is the phase of post-transplant, where high dose immunosuppressive medications influence bone architecture. Third is the reestablishment of the bone microenvironment after few years post-transplant. Last is the return of bone mineral metabolic derangements, secondary to reduced graft function [13].

The discovery of Osteoprotegerin and the receptor activator of nuclear factor kappa–B ligand (OPG/RANKL) have further advanced our knowledge and understanding of the mechanism of osteoporosis. OPG/RANKL ligands are produced by osteoblasts or bone marrow stromal cells, and have a significant role in osteoclast dedifferentiation and apoptosis [14]. A high OPG/RANKL ligand ratio inhibits bone resorption. In transgenic mice model experiments, medications like glucocorticoids have been shown to decrease the ratio of OPG/RANKL, which further increases bone resorption and reduces bone formation [15].

## 4. Overview of Risk Factors in Transplant Recipients

**Figure 2:**
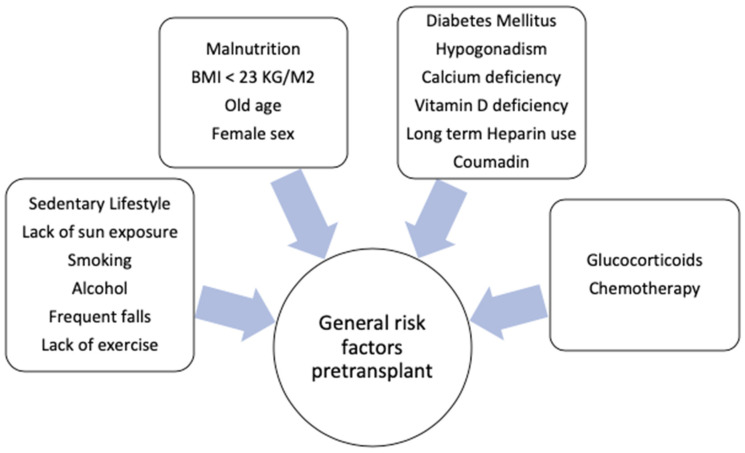
General risk factors for osteoporosis.

The general pre-transplant risk factors for osteoporosis include female sex (especially post-menopause women), BMI < 23 kg/m^2^, diabetes mellitus, malnutrition, a sedentary lifestyle, smoking, excessive alcohol, vitamin D deficiency, lack of sun exposure, hypogonadism with low estrogen and progesterone levels, number of falls, end-organ failure (including cardiac, liver and renal abnormalities) and preexisting bone mineral abnormalities [2]. Long term use of unfractionated heparin (by inhibiting osteoprotegerin and enhancing osteoclastic bone resorption) and coumadin (by inhibiting gamma-carboxylation of osteocalcin) could contribute to decreases in bone density [16]. The general risk factors for osteoporosis are illustrated in Figure 2.

### 4.1. Organ-Specific Risk Factors

#### 4.1.1. Solid Organ Transplants

##### (a) Kidney

The spectrum of bone-mineral diseases associated with chronic kidney disease is termed renal osteodystrophy. It contributes to the substantial risk of osteoporosis for transplant recipients [17]. Recent classification regrouped renal osteodystrophy, based on bone turnover, mineralization and bone volume (TMV) [18,19]. High-turnover bone disorders include mixed renal osteodystrophy and osteitis fibrosa. Low turnover is seen in adynamic bone disease [20]. Mineralization abnormalities are seen in patients with osteomalacia and mixed renal osteodystrophy [21].

The presence of underlying secondary hyperparathyroidism pre-transplant generates continued alterations in bone morphology post-transplant, leading to persistent high-turnover bone disease. Approximately 30% to 50% of patients continue to have hyperparathyroidism post-transplant [9,22,23]. No clear correlation exists between bone histomorphology and PTH (Parathyroid harmone) levels post-transplant, indicating complex interaction between multitude of factors [24]. Patients with suboptimal renal graft function post-transplant continue to suffer from renal osteodystrophy. Renal-specific risk factors associated with increased osteoporotic risk include dialysis vintage (duration of dialysis), hyperparathyroidism, chronic kidney disease (from single graft kidney and suboptimal graft function) and hypomagnesemia [12,25]. Even though osteoporotic bone manifestations are rare in patients with B2 amyloidosis; inflammatory components of amyloid, particularly IL1 and tumor necrotic factors (IL6) could result in bone-destructive changes similar to osteoporotic lesions [26,27].

**Figure 3:**
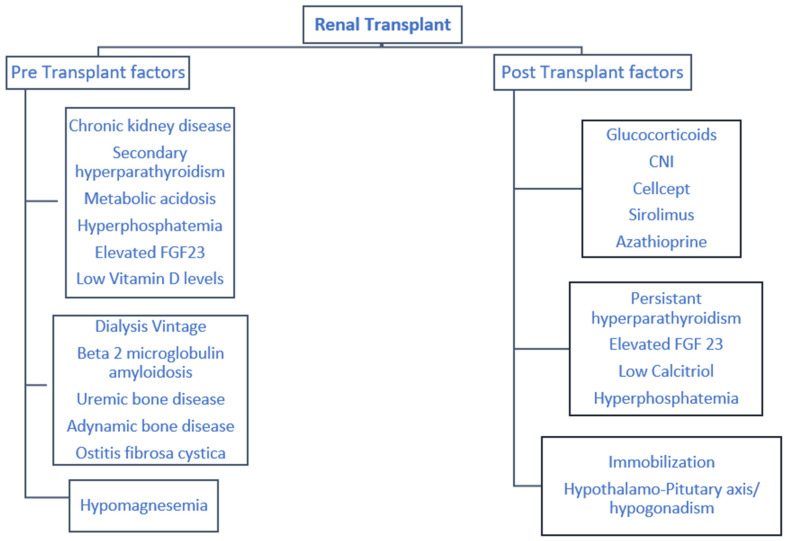
Pre and post-transplant risk factors associated with post kidney transplant osteoporosis.

Bone density loss is evident at the lumbar and femoral spine after a renal transplant. Julian et al. reported that the first six months post-renal transplant are crucial, and associated with significant osteoporotic risk [28]. Bone mineral density decreases noticeably, especially at the lumbar spine, by 4–10%. Almond et al. reported decreases in femoral neck bone density by 4% post-renal transplant [29]. The risk factors for osteoporosis prior to and after renal transplant are summarized in Figure 3.

##### (b) Liver

Bone disease following liver transplant is complex and poorly understood, given the paucity of the literature. Chronic liver disease patients typically manifest as low-turnover bone disease, secondary to osteoblast dysfunction. Their bone histomorphology revealed reduced mineralization rates and diminished collagen matrix synthesis [30]. The decreased availability of insulin-like growth factor (IGF-1) in cirrhotic patients potentiates a decline in bone mass and density, as demonstrated in rat model experiments [31]. Unconjugated hyperbilirubinemia also has inhibitory effects on osteoblast proliferation and differentiation [32].

**Figure 4:**
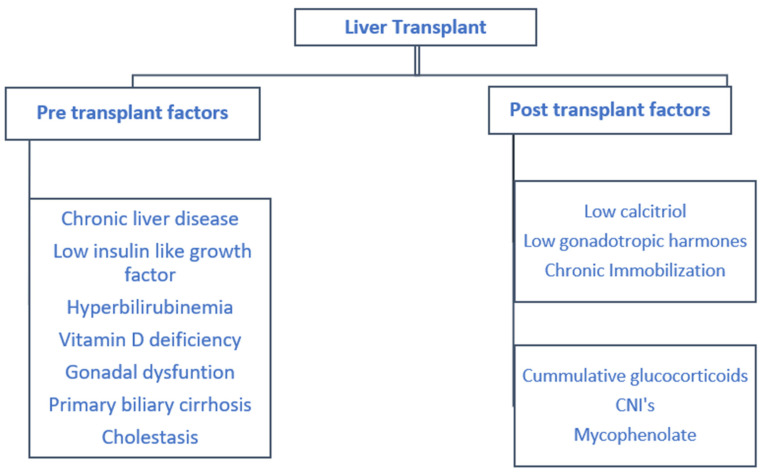
Pre- and post-transplant risk factors associated with post liver transplant osteoporosis.

Liver failure patients are particularly prone to vitamin D deficiency as a result of fat malabsorption from cholestasis, which further increases osteoclast activity [33]. Patients with primary biliary cirrhosis are particularly at risk of osteoporosis, secondary to steroid use, and post-menopausal women are affected the most [34]. The pre- and post-liver transplant risk factors are detailed in Figure 4.

##### (c) Heart

Among all cardiac patients, congestive heart failure patients experience more significant bone loss compared to others. Pisani et al. reported radiological evidence of vertebral fractures in 14% of heart failure patients awaiting transplantation [35]. Along with general risk factors contributing to low bone mass, cardiac-specific factors include patients with congestive heart failure, and renal failure associated with heart failure, leading to secondary hyperparathyroidism [36].

In the study by Shane et al. [37], a prospective cohort study of heart transplant recipients over three years reported 7.3% bone loss at the lumbar spine, compared to 10.5% at the femoral neck in the first year post-transplant. Authors concluded that the factors responsible for immediate post-transplant bone loss are a decrease in osteocalcin and serum testosterone, along with increases in bone resorption markers.

##### (d) Lung

Reduced bone mass is seen in end-stage lung disease patients, particularly in patients with COPD and cystic fibrosis. Abnormalities in osteoblasts are predominantly seen in patients with COPD [38]. Cystic fibrosis patients exhibit increased bone resorption rates compared to healthy adults, secondary to a multitude of factors, including hypogonadism, malnutrition, inflammatory cytokines, impaired calcium and vitamin D absorption from pancreatic insufficiency [39,40].

In a longitudinal study by Wang TK et al. involving lung transplant patients, pre-transplant osteopenia and osteoporosis were prevalent in 36% and 31% of patients, respectively [41]. In a prospective study by Spira et al., including lung transplant patients, bone density evaluations performed before transplant and 6–12 months after transplant suggested that moderate to severe osteopenia was evident in a significant number of patients prior to transplant [42]. Hence, strong advocacy for screening for osteoporosis prior to lung transplants prevails.

#### 4.1.2. Hematopoietic Stem Cell Transplant

Patients undergoing hematopoietic stem cell transplant (HSCT) are uniquely at risk of bone mass changes and increased frailty, secondary to nature of illness and prolonged steroid use [43]. HSCT includes bone marrow transplantation, peripheral blood stem cell transplantation, and cord blood transplantation. Certain tumors, namely myeloma and lymphoma, could lead to osteolytic bone lesions, and the subsequent generation of osteoclast-activating factors contributing to bone resorption [44]. Patients with underlying malignancies are subjected to chemo-radiation as part of treatment regimens, which predisposes them to decreased levels of IGF-1, and to subsequent hypogonadism [45]. In a study by Ebeling et al. [46], a significant percentage of women developed amenorrhea secondary to chemotherapy, even prior to HSCT.

The conditioning regimen used in preparation for HSCT, usually a combination of total body irradiation and chemotherapy, further reduce bone density, thus increasing fracture risk. Radiation causes direct damage to bone cells, with increased osteoclast activity and bone resorption [44]. Certain chemotherapy agents, like methotrexate, have direct inhibitory effects on osteoblasts; ifosfamide leads to hypophosphatemia, and long-term hypophosphatemia indirectly leads to decreased osteoblast activity. Cisplatin causes hypomagnesemia and hypocalcemia with excessive PTH, leading to bone resorption [44].

Studies comparing the incidence and severity of bone loss among autologous and allogenic HSCT patients have demonstrated mixed results. Multiple studies have demonstrated increased bone density loss in allogenic stem cell transplant patients, compared to autologous patients [47,48]. Studies by Pundol et al. [49] and Lin et al. [50] have revealed the higher risk of fractures among autologous compared to allogenic patients. A significant percentage of patients with allogeneic bone marrow transplants develop graft-versus-host disease (GVHD), and are routinely treated with high dose steroids [51,52], predisposing them to a reduction in bone density.

The risk factors for osteoporosis associated with heart, lung and bone marrow transplants are summarized in Table 1.

### 4.2. Post-Transplant Medications

#### 4.2.1. Glucocorticoids

Glucocorticoids (GC) are commonly used after transplantation to reduce the risk of graft rejection. Glucocorticoids harm skeletal health by reducing bone formation and potentiating bone destruction, leading to decreases in bone density [53]. Glucocorticoid’s effect is seen especially at the trabecular level of vertebral bone. Glucocorticoids induce accelerated bone microarchitectural changes in the first six months after transplant [28]. They contribute to osteoblast and osteocyte apoptosis by reducing levels of type 1 collagen, IGF-1 and osteocalcin. Serial bone biopsies at 22 days and 160 days after transplantation in a study suggested that glucocorticoids were associated with impaired osteoblast formation [53]. Glucocorticoids also promote osteoclast activity by increasing the levels of RANK ligand, thereby decreasing the ratio of osteoprotegerin to RANK ligand [54]. Glucocorticoids have the indirect effect of promoting intestinal calcium absorption, thereby augmenting secondary hyperparathyroidism [55]. Glucocorticoids indirectly perpetuate hypogonadism, leading to bone loss. Steroid myopathy has been reported after high doses post-transplant, leading to vertebral imbalance from gravitational forces and increased fracture risk [56].

One of the debilitating musculoskeletal complications after renal transplant is osteonecrosis. These are typically reported 3 to 6 months post-transplant at weight-bearing surfaces, especially at the femoral head, leading to bone collapse [57]. Steroid minimization protocols are being used more often to decrease the adverse effects of long-term high dose steroids [58].

#### 4.2.2. Calcineurin Inhibitors

Calcineurin inhibitors (CNI), cyclosporine and tacrolimus facilitate immunosuppression by inhibiting T lymphocytes and IL2 production [59]. CNI cause hypomagnesemia, leading to elevated levels of parathyroid hormone. Secondary hyperparathyroidism post-transplant will accelerate high-turnover bone disease, potentiating increased osteoporotic fracture risk [23].

The effect of cyclosporine on bone density is unclear, as it is typically used in combination with glucocorticoids. In the murine experimental models by Schlosberg and Movsowitz, cyclosporine stimulates osteoblast and osteoclasts activity. However, osteoclastic activity dominates, leading to bone resorption [60]. The human effects of cyclosporine are ill-defined [61]. Tacrolimus induces severe trabecular bone loss with high-turnover bone disease, as studied in rat experimental models [62], however the literature on human effects is minimal. In studies by Monegal et al., in liver transplant recipients, tacrolimus was associated with a higher femoral neck bone density as compared to cyclosporine, two years post-transplant [63].

#### 4.2.3. Other Immunosuppressants

Other immunosuppressive medications used post-transplantation include mycophenolate, sirolimus and azathioprine. Limited data is available regarding the effects of these medications on bone mineral metabolism post-transplant. Previous rodent experiments did not show any effect of the above-mentioned immunosuppressive medications on bone volume [64]. Studies by Singha et al. have helped understand the effects of sirolimus on bone metabolism, showing it could interfere with the proliferation and differentiation of osteoblast [65]. In mouse models, everolimus demonstrated inhibitory effects on osteoclast activity [66].

### 4.3. Endocrine and Mineral Metabolism

#### 4.3.1. PTH

The optimal PTH levels following renal transplant remain unclear. With improvements in kidney function, the levels of calcium, phosphorous and vitamin D relatively improve post-transplant [67]. The levels of parathyroid hormone usually decrease by half at around six months post-renal transplantation. However, in nearly 50% of the patients, the levels remain elevated one to two years post-transplant [68]. Prolonged renal failure before transplantation, dialysis vintage, high levels of PTH and elevated alkaline phosphatase levels are shown to be associated with persistent post-transplantation hyperparathyroidism [69]. In a single-center study done by Perrin et al., involving kidney transplant recipients, persistent hyperparathyroidism at three months was associated with a remarkable increase in fracture risk [70]. In a recent analysis done by Lou I et al., involving kidney transplants recipients, persistent hyperparathyroidism was associated with reduced graft survival [68]. Persistently elevated parathyroid levels could perpetuate calcium deposition in the soft tissues, blood vessels and heart, leading to hypophosphatemia hypercalciuria, accelerated atherosclerosis, and risk of calciphylaxis [71].

#### 4.3.2. Phosphorous

Post-transplant hypophosphatemia is a fairly common occurrence, likely related to uncontrolled hyperparathyroidism and altered mineral metabolism in end-stage kidney disease, which continues into the early post-transplant period. Hypophosphatemia is associated with derangement in bone mineralization and decreased osteoblast activity, and further potentiates the development of rickets and osteomalacia [67].

#### 4.3.3. Calcitriol

Low serum vitamin D levels are frequently found in the immediate post-transplant period, and also in long-term graft recipients. In about one third of post-renal transplant recipients, even after successful engraftment, vitamin D levels tend to be low when compared to healthy individuals [72]. Calcitriol levels fall in the post-transplant period, which is attributed to the use of steroids and other immunosuppressive medications. Steroids inhibit the activity of one alfa hydroxylase, potentially leading to a reduction in calcitriol levels [73]. Persistent 25-OHD deficiencies are associated with hypocalcemia and hypophosphatemia. Furthermore, low 25-OHD levels, possibly a result of the immune-modulatory mechanism, could be associated with poor graft outcomes, including an increased risk of acute cellular rejection [74].

#### 4.3.4. Hypothalamic–Pituitary–Gonadal (HPG) Axis Post-Transplant

Following solid organ transplantation, the HPG axis’ physiological functions resume in the majority of patients. Many heart transplant recipients’ testosterone levels fall immediately post-transplantation, and normalize within the first year after the transplant [75]. In the majority of liver transplant patients, the physiological function of sex steroids will be resumed with the normalization of the HPG axis [76]. Uremia-induced hyperprolactinemia is significantly corrected post-renal transplant in many men and premenopausal women [77]. No concurrent data is available on the HPG axis in lung transplant patients.

### 4.4. Immobilization

Given the prolonged wait list times for solid organ transplants, progression of disease activity, significant malnutrition and immobilization are all quite rampant. Immobilization could further persist in the post-transplant period if adequate steps are not taken [78]. Immobilization may add to the adverse effects on bone mineral metabolism, transforming to significant bone disease. Additionally, in studies by Kwan et al., the severity of bone loss was correlated with the length of hospital stay post-transplantation [79].

## 5. Evaluation and Diagnosis

Screening for pre-transplant risk factors would be beneficial in predicting the post-transplant course. History of bone pain, vitamin D deficiency, hypogonadism, previous fractures, steroid use and secondary hyperparathyroidism predispose the patient to osteoporosis post-transplantation. A sedentary lifestyle, malnutrition, and tobacco and alcohol usage need to be addressed pre-transplantation to help overcome bone mineral abnormalities post-transplant [25]. The risk tends to be higher in the first six months to one year post-transplant. However, in later months, with the reduction in glucocorticoid dose, the loss of bone mass declines.

The KDIGO (Kidney Disease Improving Global Outcomes) 2017 guidelines for post-kidney transplant patients recommend checking serum calcium and phosphate weekly post-transplant, until they are more stable. PTH and Vitamin D should be checked based on renal function every three to six months post-renal transplant [80]. In post-liver transplant patients, it is recommended to check the serum calcium, phosphate levels, vitamin D levels, thyroid function studies, parathyroid hormone levels and testosterone levels in men, and estrogen levels in women, annually [81]. The evaluation and diagnosis are summarized in Table 2.

### 5.1. Fracture Risk Assessment (FRAX)

FRAX is a clinical tool for predicting the 10-year fracture risk in the general population. It implements a questionnaire including age, sex, weight, smoking and alcohol history, previous fractures, glucocorticoid use, family history of fractures and bone density [21]. Recently, in a single-center study by Naylor et al. [82] including kidney transplant patients, FRAX was shown to predict fracture risk; however, we need larger cohort studies in order to validate the results of FRAX use for transplant recipients before wide-spread use is implemented.

### 5.2. DEXA Scan

DEXA is a noninvasive, relatively accurate test that assists in predicting osteoporosis and fracture risk. Bone density measured by DEXA after kidney transplants indicated significant bone loss at the lumbar spine in about 40% of patients, however no noticeable changes were observed at the femoral neck six months post-transplant [17]. Bradenburg et al. measured lumbar bone mineral density in kidney transplant recipients utilizing DEXA scans, it decreased by −0.6% ± 1.9% annually [83]. The study by Akaberi et al., involving kidney transplant recipients with osteopenia and osteoporosis at the hip, reported an increased risk of fracture for these patients compared to non-transplant patients with osteopenia and osteoporosis, analyzed by DEXA [84]. However, a DEXA scan, as a predictive marker for fracture risk, is not without flaws. DEXA scans pose challenges in differentiating cortical vs trabecular bone in post-renal transplant patients, and cannot differentiate vascular from soft tissue calcification [85,86]. The predictive value of DEXA scans for lumbar fractures is currently uncertain, particularly for post-kidney transplant recipients. Based on the available data, KDIGO 2017 has recommended bone mineral density testing in renal transplant patients at substantial risk of osteoporosis [87].

DEXA provides a two-dimensional measurement of bone density, generating difficulties in understanding bone disease quality and strength. Complex bone histomorphology post-transplant necessitates advanced testing beyond DEXA. High-resolution quantitative computed tomography (HRCT) guides us in understanding the density and microarchitecture of cortical and trabecular regions separately, and is currently used for research purposes, given its high cost and non-generalizability of the results [88]. Further additional information is needed before HRCT is standardized to the post-transplant population.

### 5.3. Bone Biopsy

Bone biopsy is an invasive, gold standard procedure for evaluating bone abnormalities post-transplantation. KDIGO recommends considering bone biopsies post-transplant, for patients with persistent bone pain and who are at risk of fractures, to help guide treatment [89]. In most transplant patients, there is a slight increase in osteoclast activity, a decrease in osteoblast function, and an alteration in bone dynamics [85,90]. In post-renal transplant patients, uremia-related bone changes are resolved; however, hyperparathyroidism changes persist, making bone biopsy results difficult to interpret [91]. Most centers lack expertise in the interpretation of results from post-transplant patients. Follow-up biopsies looking at dynamic changes might be challenging, this being an invasive procedure. Bone histomorphometric changes could be different at different sites, including the radius, femur and hip, which could make results difficult to generalize [92].

## 6. Management

General measures that help mitigate bone loss (Table 3) can be applied to transplant patients. Calcium 1000 mg/day and Vitamin D 800 IU/day supplementation, regular weight-bearing exercises for 30 mins three times a week, smoking cessation, and early mobilization post-transplant are some measures that support bone loss prevention [89]. Evaluation and correction of underlying causes, reducing negative calcium balance, early mobilization post-transplant and avoidance of medications with negative impacts on bone metabolism could potentially minimize osteoporotic risk. Identifying patients at increased risk of post-transplant osteoporosis will be beneficial, as lower bone mineral density is a potential risk factor for fractures [93]. ESRD patients will subsequently require evaluation and management of underlying renal osteodystrophy [94].

### 6.1. Renal Transplant

The optimal management strategies to prevent osteoporosis in patients at high risk of fractures are not clear. Calcium and vitamin D supplementation in patients with deficiencies could be an initial step in the prevention of osteoporosis. Studies with active vitamin D analogs have reported the prevention of bone loss at the femoral and lumbar regions, but have not been sufficiently powered to determine fracture outcomes [73,95]. The risk of associated hypercalcemia and hypercalciuria with excessive calcium and vitamin D supplementations should also be considered [96]. Some authors consider prophylactic therapies with anti-resorptive medications for patients with osteopenia and a high risk of fracture. However, no strong evidence exists regarding the benefits of anti-resorptive therapies, including bisphosphonates and denosumab, in preventing fractures [97,98].

A stepwise management approach should be considered in the treatment of osteoporosis among renal transplant recipients. The treatment of patients with osteoporosis prior to, or at the time as, transplantation is similar to osteoporosis management for chronic kidney disease (CKD) patients. Management of persistent hyperphosphatemia and secondary hyperparathyroidism should be considered initially [99]. In patients with no evidence of low-turnover bone disease and GFR > 30 mL/min/1.732, antiresorptive therapies, such as bisphosphonates, could be considered [100,101,102]. In the study by Coco et al. [103], pamidronate along with calcium and calcitriol was associated with the preservation of vertebral bone mineral density, but also with the increased risk of low bone turnover. Denosumab, a monoclonal antibody against the NF-kb ligand, has been shown to preserve bone density and decrease the risk of fractures in women. Denosumab can be considered as an alternative to bisphosphonates in patients with low GFR [98,104]. However the rebound risk of spontaneous vertebral fractures after discontinuation of denosumab, along with the hypocalcemia risk, should be taken into consideration [105,106]. Limited data are available regarding the use of recombinant human PTH (rPTH) in osteoporosis treatment among renal transplant recipients. The treatment options are summarized in Table 4.

### 6.2. Other Transplants

Considering that transplant recipients are more vulnerable to fractures in the immediate post-transplant period, especially while on glucocorticoids, preventive therapy is recommended for all heart, liver, lung and stem cell transplant recipients, up to one year post-transplant. Bisphosphonates attenuate glucocorticoid-induced bone loss, reduce the incidence of fractures in post-transplant recipients, and are frequently administered for the prevention and treatment of osteoporosis [107,108,109,110,111,112]. In a meta-analysis comparing bisphosphonates or vitamin D analogs to a placebo after solid organ (liver, heart and kidney) transplantation, a substantial reduction in fracture rates, including vertebral and total fracture rates, one year post-transplant was demonstrated [113]. However, bisphosphonates should be used with caution in pre-menopausal women and in those with underlying renal or allograft dysfunction. Duration of therapy is typically determined by multiple factors, including cumulative glucocorticoid dose post-transplant, bone mineral density measurements and fracture risk [114]. Other treatments, including calcitriol [115,116] and hormone replacement therapies in selective patients with hypogonadism [117], have been efficacious in preventing bone loss following transplantation. There is no available data regarding Denosumab for the prevention of bone loss after heart, lung or marrow transplants. In a small study involving liver transplant recipients, Denosumab was associated with an increase in bone density, however further, larger cohort studies are needed to validate the results [118]. Teriparatide (rPTH) has been studied only in renal transplant recipients [119].

## 7. Conclusions

Post-transplantation bone disease is complex, and management should focus on correcting the metabolic abnormalities associated with bone diseases. The primary goal is to identify high-risk individuals and implement preventive strategies to help minimize fracture risk. Fractures are associated with increased mortality and morbidity. Multiple medications have been shown to reduce bone loss post-transplant; however, no substantial data is available regarding clinical outcomes. Therefore, there is a significant need for high-quality, evidence-based studies, in order to develop treatment modalities that could help mitigate fracture risks following organ transplantation. Even though DEXA scans help predict bone density and fractures, significant limitations exist in predicting fracture risk in renal transplant recipients. Hence more sophisticated imaging modalities, to conveniently identify high-risk individuals, are further warranted.

## Figures and Tables

**Table 1:** Risk factors associated with Heart/lung and bone marrow transplant. COPD – Chronic Obstructive Pulmonary Disease, GVHD – Graft Versus Host Disease.

	Heart	Lung	Bone Marrow
Organ-specific risk factors	Congestive heart failure Low Vitamin D levels Co-existing renal failure Hypogonadism Long term heparin use	COPD Cystic Fibrosis Inflammatory cytokines Prolonged glucocorticoid use	Leukemia/Lymphoma Hypogonadism Conditioning regimen (Melphalan, Busulphan) Total body irradiation Chemotherapy GVHD

**Table 2:** Evaluation of patients with post-transplant osteoporosis. (H/O – History Of).

Evaluation
*(a) History/Physical*
H/O recent fracture
Back pain/bone pain
Family H/O osteoporosis
Exercise
Smoking cessation
Alcohol cessation
*(b) Serologies*
Serum calcium level
Serum phosphorous level
Serum parathyroid level
Estrogen levels
Progesterone level
TSH level
*(c) FRAX*
Predicts 10% fracture risk
*(d) DEXA-*scan Annually

**Table 3:** General measures to mitigate bone loss.

General Measures
Stratification of high-risk individuals Regular weight-bearing exercise Smoking cessation Early mobilization post-transplant Shorter steroid course post-op Avoidance of medication with negative calcium balance Calcium 1000 mg/day, Vitamin D 500 IU/day Evaluation and Correction of underlying cause

**Table 4:** Treatment options for post-transplant osteoporosis.

	Renal Transplant	Other Transplants
Treatment options	Management of secondary hyperparathyroidism Management of hyperphosphatemia Bisphosphonates (limited to patients with eGFR > 30 mL/min, risk of adynamic bone disease) Denosumab (risk of hypocalcemia) Teriparatide	Bisphosphonates Calcitriol Hormone replacement therapy (only for patients with hypogonadism) Denosumab
